# Fully Developed Opposing Mixed Convection Flow in the Inclined Channel Filled with a Hybrid Nanofluid

**DOI:** 10.3390/nano11051107

**Published:** 2021-04-25

**Authors:** Xiangcheng You, Shiyuan Li

**Affiliations:** College of Petroleum Engineering, China University of Petroleum-Beijing, Beijing 102249, China; lishiyuan1983@cup.edu.cn

**Keywords:** hybrid nanofluid, mixed convection, inclined channel, flow reversal

## Abstract

This paper studies the convective heat transfer of a hybrid nanofluid in the inclined channel, whose walls are both heated by the uniform heat flux. The governing ordinary differential equations are made nondimensional and solved analytically, in which explicit distributions of velocity, temperature and pressure are obtained. The effects of flow reversal, wall skin friction and Nusselt number with the hybrid nanofluid depend on the nanoparticle volume fractions and pressure parameters. The obtained results indicate that the nanoparticle volume fractions play a key role in delaying the occurrence of the flow reversal. The hybrid nanofluids hold more delayed range than conventional nanofluids, which is about 2.5 times that of nanofluids. The calculations have been compared with the base fluid, nanofluid and two kinds of hybrid models (type II and type III). The hybrid model of type III is useful and simplified in that it omits the nonlinear terms due to the interaction of different nanoparticle volumetric fractions, with the relative error less than 3%. More results are discussed in the results section below.

## 1. Introduction

Research on mixed convection inducted flow is increasingly interested in many engineering applications, such as in heat exchangers, chemical processing equipment, transport of heated or cooled fluids, solar power collectors, microelectronic cooling and so on. Furthermore, the mixed convection flow through a channel has received a great deal of attention [1,2,3,4,5] in literature. However, most of the previous studies have focused on the horizontal or vertical configuration and mixed convection in inclined geometries has been studied less. Thus, it is worthwhile to explore the flow on the inclined flat plate at various angles which is often come across in engineering devices, such as solar water heaters, inclination/acceleration sensors and so on. Bohne et al. [6] investigated superposed free and forced convection in an internally heated concentric annulus in vertical, inclined and horizontal position with experiments. Lavine [7] presented an exact solution of fully developed, laminar flow between inclined parallel plates with a uniform wall heat flux boundary condition. Wang [8] studied numerically with fully developed opposing mixed convection in an inclined channel that had discrete heating on the bottom and was insulated on the top. Barletta et al. [9] researched analytically the fully developed laminar mixed convection with viscous dissipation in an inclined channel with prescribed wall temperatures. Aydin et al. [10] investigated MHD mixed convective heat transfer flow about an inclined plate. Cimpean [11] studied the steady fully developed mixed convection flow of a nanofluid in a channel filled with a porous medium. You et al. [12] presented analysis of fully developed opposing mixed convection flow in an inclined channel filled by a nanofluid. Goyal et al. [13] examined numerically natural convective boundary layer flow of a nanofluid past a heated inclined plate in the presence of magnetic field and found that the thermal boundary layer thickness increased with strengthening the value of inclination angle parameter. Rafique et al. [14] studied numerically on micropolar nanofluid flow over an inclined surface by means of Keller-Box method. Khademi et al. [15] studied numerical analysis of mixed convection flow of nanofluid over an inclined flat plate embedded in a porous medium in the presence of a transverse magnetic field. Anuar et al. [16] presented work explored the heat transfer and boundary layer flow of a hybrid nanofluid past an inclined stretching/shrinking sheet with suction and buoyancy force effects.

It is well-known that nanofluids have been applied to problems with the thermal properties of heat transfer fluids. The nanofluids were first researched by Choi and Eastman [17]; they are kinds of suspended nanoparticles in the base fluid, such as ethylene glycol, oil or water. The properties of the nanofluids are higher than the base fluid, such as viscosity, diffusion coefficient, heat transfer rate and thermal conductivity [18,19,20,21,22]. Nanofluids can be used in microchip cooling, nuclear reactor, industrial cooling, sensing, drug delivery, nanomedicine, the oil recovery process and so on [23,24]. However, hybrid nanofluids are composed of two different nanoparticles dispersed in the base fluid, which have better thermophysical properties and rheological behavior along with improved heat transfer properties [25,26,27]. In recent years, many scientists and researchers have been attracted to investigating real-world heat transfer problems with hybrid nanofluids [28,29,30]. Obviously, it is necessary to study the mechanisms of hybrid nanofluids that contribute to the heat transfer enhancement.

Motivated and based on the literature discussed above, the main aim of this paper is to study the fully developed mixed convection flow in the inclined channel filled with a hybrid nanofluid, which employs simply homogeneous model proposed by Maïga et al. [31]. In this study, hybrid nanofluid is formed by suspending two different nanoparticles, which are copper and alumina, in the base fluid. The governing equations with boundary conditions are solved analytically that have never been reported before based on the literature survey. The model analyses the hybrid nanofluid behavior by comprising the nanoparticles solid volume fractions, which using two kinds of hybrid models (type II and type III). The result shows that the hybrid model of type III is useful and simplified that omits the nonlinear terms due to the interaction of different nanoparticle volumetric fractions. Besides, the effects of the main physical parameters are discussed respectively, such as the nondimensional pressure parameter $P_{1},P_{2}$, the nanoparticle volume fractions $\phi_{1},\phi_{2}$, the velocity profile U(Y), the temperature profile $\theta -\theta_{b}$, the average wall friction $\bar{C_{f}Re}$ and the average Nusselt number $\bar{Nu}$; these are illustrated graphically. The hybrid nanofluids hold better thermophysical properties than conventional nanofluids based on the results.

## 2. Mathematical Model

**Figure 1:**
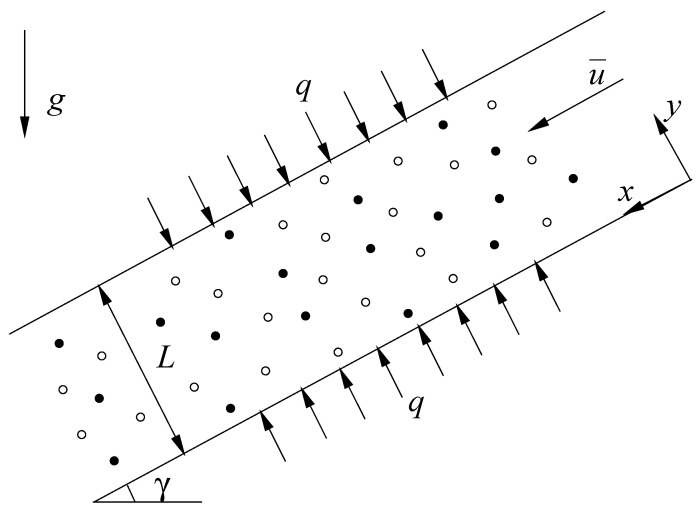
Physical configuration and coordinate system.

Consider the steady mixed convection flow, which is driven by a buoyancy force and an external pressure gradient between two paralleled long inclined plane walls filled with a hybrid nanofluid and separated by a distance *L*. Figure 1 shows the sketch of system and the coordinate axes, where *x* and *y* axes are measured along the lower plane of the channel oriented in the downward direction, *y* axis is in the normal to the lower plane, *q* is the constant wall heat flux, *g* is the acceleration due to gravity and γ is the inclination angle of the channel. The velocity field is given in the case by v(u,0), then the continuity equation reduces to ∂u/∂x=0 and implies u=u(y). Following Lavine [7] and using the hybrid nanofluid model, the momentum balance and energy equations according to the Boussinesq approximation are written by

(1) $$\frac{\partial p}{\partial x}=-{\left(\rho \beta \right)}_{hnf}\;g\left(T-T_{0}\right)\sin\gamma +\mu_{hnf}\frac{d^{2}u}{dy^{2}},$$

(2) $$\frac{\partial p}{\partial y}={\left(\rho \beta \right)}_{hnf}\;g\left(T-T_{0}\right)\cos\gamma ,$$

(3) $$u\frac{\partial T}{\partial x}=\alpha_{hnf}\frac{\partial^{2}T}{\partial y^{2}},$$

subject to the boundary conditions

(4) $$u\left(0\right)=0,\;u\left(L\right)=0,\;-{\left.\left(\frac{\partial T}{\partial y}\right)\right|}_{y=0}={\left.\left(\frac{\partial T}{\partial y}\right)\right|}_{y=L}=1.$$

The mass flow rate is assumed as a prescribed quantity of this channel flow study, then the following average fluid velocity in the section will be prescribed as

(5) $$\bar{u}=\int_{0}^{L}u\left(y\right)dy.$$

Here *u* is the velocity component along the *x* axis, $\bar{u}$ is the average velocity, *T* is the temperature of the hybrid nanofluid, $T_{0}$ is the constant reference temperature, *p* is the fluid thermodynamic pressure and *g* is the gravitational acceleration. The physical quantities in Equations (1)–(3) are $\phi_{1},\phi_{2}$ is the nanoparticle volume fractions, $\beta_{f},\beta_{n1},\beta_{n2}$ are the coefficients of thermal expansion of the base fluid and nanofluid respectively, $\rho_{f},\rho_{n1},\rho_{n2}$ are the densities of the base fluid and nanofluid, $\mu_{hnf}$ is the viscosity of the hybrid nanofluid and $\alpha_{hnf}$ is the thermal diffusivity of the hybrid nanofluid, $\mu_{f}$ is the dynamic viscosity of the base fluid and its expression has been proposed by Brinkman [32], $k_{hnf}$ is the thermal conductivity of the hybrid nanofluid, $k_{f},k_{n1},k_{n2}$ are the thermal conductivities of the base fluid and nanofluid, ${\left(\rho C_{p}\right)}_{hnf}$ is the heat capacitance of the hybrid nanofluid. Note that the expression (5) is restricted to spherical nanoparticles where it does not account for other shapes of nanoparticles. The thermophysical properties of the base fluid, nanofluid and hybrid nanofluid are given in Table 1, Table 2, Table 3 and Table 4, referring to references [33,34,35,36,37].

Introduce the following dimensionless variables

(6) $$\begin{array}{c} X=\alpha_{f}x/\left(\bar{u}L^{2}\right),\;Y=y/L,\;U\left(Y\right)=u/\bar{u},\\ \theta \left(X,Y\right)=\left(T-T_{0}\right)/\left(qL/k_{f}\right),\\ P\left(X,Y\right)=\left[p-\rho_{f}g\left(x\sin\gamma -y\cos\gamma \right)\right]/\left(\mathrm{Pr}\rho_{f}{\bar{u}}^{2}\right), \end{array}$$

where $\alpha_{f}$ is the thermal diffusivity of the base fluid, $\nu_{f}$ is the kinematic viscosity of the base fluid, $\mathrm{Pr}=\nu_{f}/\alpha_{f}$ is the Prandtl number. Substituting variables (6) into Equations (1)–(3), the following dimensionless equations are obtained

(7) $$\frac{\partial P}{\partial X}=-P_{1}\frac{{\left(\rho \beta \right)}_{hnf}}{{\left(\rho \beta \right)}_{f}}\theta +\frac{\mu_{hnf}}{\mu_{f}}\frac{d^{2}U}{dY^{2}},$$

(8) $$\frac{\partial P}{\partial Y}=P_{2}\frac{{\left(\rho \beta \right)}_{hnf}}{{\left(\rho \beta \right)}_{f}}\theta ,$$

(9) $$U\frac{\partial \theta }{\partial X}=\frac{\alpha_{hnf}}{\alpha_{f}}\frac{\partial^{2}\theta }{\partial Y^{2}},$$

subject to the boundary conditions

(10) $$U\left(0\right)=0,\;U\left(1\right)=0,\;-{\left(\frac{\partial \theta }{\partial Y}\right)}_{Y=0}={\left(\frac{\partial \theta }{\partial Y}\right)}_{Y=1}=1,$$

along with the mass flux conservation relation

(11) $$\int_{0}^{1}UdY=1.$$

Here $P_{1}=Gr\sin\gamma /Re$ and $P_{2}=Gr\cos\gamma /\left(\mathrm{Pr}Re^{2}\right)$ are nondimensional pressure parameters, $Gr=g\beta_{f}qL^{4}/\left(k_{f}\nu_{f}^{2}\right)$ is the Grashof number and $Re=\bar{u}L/\nu_{f}$ is the Reynolds number.

Integrating Equation (9) over the channel cross-section, making use of Equation (11) and the boundary conditions (10) for temperature distribution θ, and considering the constant heat flux distribution of *x*-direction at the walls, the following relation can be obtained

(12) $$\frac{\partial \theta }{\partial X}=2\frac{\alpha_{hnf}}{\alpha_{f}}.$$

Differentiating Equation (7) with *Y* and Equation (8) with *X* respectively, and taking into account (12), then equating them, we obtain

(13) $$-P_{1}\frac{{\left(\rho \beta \right)}_{hnf}}{{\left(\rho \beta \right)}_{f}}\frac{\partial \theta }{\partial Y}+\frac{\mu_{hnf}}{\mu_{f}}\frac{d^{3}U}{dY^{3}}=2\frac{\alpha_{hnf}}{\alpha_{f}}\frac{{\left(\rho \beta \right)}_{hnf}}{{\left(\rho \beta \right)}_{f}}P_{2}.$$

Differentiating this equation with *Y* once again, we obtain

(14) $$\frac{\mu_{hnf}}{\mu_{f}}\frac{d^{4}U}{dY^{4}}=P_{1}\frac{{\left(\rho \beta \right)}_{hnf}}{{\left(\rho \beta \right)}_{f}}\frac{\partial^{2}\theta }{\partial Y^{2}}.$$

Making use of Equations (9) and (12), we finally obtain

(15) $$\frac{\mu_{hnf}}{\mu_{f}}\frac{d^{4}U}{dY^{4}}=2P_{1}\frac{{\left(\rho \beta \right)}_{hnf}}{{\left(\rho \beta \right)}_{f}}U.$$

This equation can be solved analytically for the velocity distribution *U*, the temperature distribution θ and the pressure distribution *P* can be determined as well. The analytical solution of Equation (15) can be obtained by using the computational softwares such as MATHEMATICA or MAPLE.

Using the software MATHEMATICA and taking account of the boundary conditions (10), the analytical solutions of the velocity distribution *U*, the temperature distribution θ and the pressure distribution *P* can be given explicitly as

(16) U(Y)=a[sinh(mY)+sin(mY)]+b[cosh(mY)−cos(mY)]+csin(mY),

(17) $$\begin{array}{cc} \theta \left(X,Y\right) & =\frac{2}{m^{2}}\{a\left[\sinh\left(mY\right)-\sin\left(mY\right)\right]+b[\cosh\left(mY\right)\\ & +\cos\left(mY\right)]-c\sin\left(mY\right)\}-\frac{2P_{2}}{P_{1}}\frac{\alpha_{hnf}}{\alpha_{f}}Y+2\frac{\alpha_{hnf}}{\alpha_{f}}X+A, \end{array}$$

(18) $$\begin{array}{cc} P\left(X,Y\right) & =\frac{{\left(\rho \beta \right)}_{hnf}}{{\left(\rho \beta \right)}_{f}}\{\frac{2P_{2}}{m^{3}}\{a\left[\cosh\left(mY\right)+\cos\left(mY\right)\right]\\ & +b[\sinh(mY)+\sin(mY)]+c\cos(mY)\}\\ & +P_{2}\left(2\frac{\alpha_{hnf}}{\alpha_{f}}XY+AY-\frac{P_{2}}{P_{1}}\frac{\alpha_{hnf}}{\alpha_{f}}Y^{2}\right)-P_{1}\left(AX+\frac{\alpha_{hnf}}{\alpha_{f}}X^{2}\right)+B\}, \end{array}$$

where *A* and *B* are the constants, whose values are dependent on the given values of $P_{1}$ and $P_{2}$, and they must satisfy the boundary conditions for θ and *P*. The parameters *a*, *b*, *c* and *m* are given as

(19) $$\begin{array}{c} a=\frac{\left[m-c+c\cos(m)][\cosh(m)-\cos(m)\right]}{-2[\cosh(m)\cos(m)-1]}+\frac{c\sin(m)[\sinh(m)-\sin(m)]}{-2[\cosh(m)\cos(m)-1]},\\ b=\frac{\left[m-c+c\cos(m)][\sinh(m)+\sin(m)\right]}{2[\cosh(m)\cos(m)-1]}+\frac{c\sin(m)[\cosh(m)-\cos(m)]}{2[\cosh(m)\cos(m)-1]},\\ c=m\left(\frac{1}{2}-\frac{P_{2}}{P_{1}}\frac{\alpha_{hnf}}{\alpha_{f}}\right),\;m^{4}=2P_{1}\frac{{\left(\rho \beta \right)}_{hnf}}{{\left(\rho \beta \right)}_{f}}\frac{\mu_{f}}{\mu_{hnf}}. \end{array}$$

Once the values of $P_{1}$ and $P_{2}$ are artificially prescribed, the analytical solutions of *U*, θ and *P* can be fully determined. Note that the analytical solution for the horizontal case $\left(P_{1}=0\right)$ can be obtained by expanding the above solutions for small *m*, or more simply, by solving the Equations (7)–(9) by setting $P_{1}=0$. Noting that for $\phi_{1}=\phi_{2}=0$, the solutions are reduced to those of Lavine [7].

The physical quantities of practical interest in this problem are the wall friction $C_{f}\;Re$ and the Nusselt number Nu, they are given by

(20) $$C_{f}\;Re=\frac{\tau_{w}}{\frac{1}{2}\rho_{f}{\bar{u}}^{2}}\frac{\bar{u}L}{\nu_{f}},\;Nu=\frac{2qL}{k_{f}\left(T_{w}-T_{b}\right)},$$

where $T_{w}$ is the wall temperature, $T_{b}$ is the bulk temperature. $\tau_{w}$ is the wall shear stress which is defined by

(21) $$\tau_{w}=\pm \mu_{nf}\frac{\partial u}{\partial y}{|}_{0,L},$$

where ± signs correspond to the bottom and top walls, respectively.

Substituting Equations (6) and (21) into Equation (20), we obtain

(22) $$C_{f}\;Re=\left\{\begin{array}{cc} 2m\frac{\mu_{hnf}}{\mu_{f}}\left(2a+c\right) & \mathrm{at}\;Y=0,\\ -2m\frac{\mu_{hnf}}{\mu_{f}}\{a\left[\cosh\left(m\right)+\cos\left(m\right)\right]\\ +b[\sinh(m)+\sin(m)]+c\cos(m)\} & \mathrm{at}\;Y=1, \end{array}\right.$$

and

(23) $$Nu=\frac{2qL}{k_{f}\left(T_{w}-T_{b}\right)}=\frac{2}{\left(\theta_{w}-\theta_{b}\right)},$$

where

(24) $$\theta_{b}=\int_{0}^{1}U\theta dY\;\mathrm{and}\;\theta_{w}=\theta \left(Y=0,1\right).$$

The average of the top and bottom wall friction is also of interest. It is given by:

(25) $$\begin{array}{c} \bar{C_{f}Re}=\frac{1}{2}\left[C_{f}Re\left(Y=0\right)+C_{f}Re\left(Y=1\right)\right]\\ \;=\frac{\mu_{hnf}}{\mu_{f}}\frac{m^{2}\left[\cos\left(m\right)-\cosh\left(m\right)-\sin\left(m\right)\sinh\left(m\right)\right]}{\cos(m)\cosh(m)-1}. \end{array}$$

Thus, the average wall friction ($\bar{C_{f}Re}$) is seen to be independent of parameter $P_{2}$.

Similarly, the average of the top and bottom Nusselt number is given by:

(26) $$\begin{array}{cc} \bar{Nu} & =\frac{1}{2}\left[Nu\left(Y=0\right)+Nu\left(Y=1\right)\right]\\ & =\frac{1}{\theta_{w}{|}_{Y=0}-\theta_{b}}+\frac{1}{\theta_{w}{|}_{Y=1}-\theta_{b}}. \end{array}$$

Furthermore, the average Nusselt number ($\bar{Nu}$) is dependent of parameter $P_{2}$.

## 3. Results and Discussion

To comprehend the current problem, the physical influence of the governing parameters, such as the pressure parameters $P_{1},P_{2}$, the nanoparticle volume fractions $\phi_{1},\phi_{2}$, the velocity profile U(Y), the temperature profile $\theta -\theta_{b}$, the average wall friction $\bar{C_{f}Re}$ and the average Nusselt number $\bar{Nu}$ are illustrated graphically. The flow reversal occurs to the upper wall satisfies ${\left.(dU/dY)\right|}_{Y=1}=0$, or $C_{f}{\;Re|}_{Y=1}=0$. The following constraint relationship of $P_{1}$ and $P_{2}$ based on Equation (22) can be obtained as

(27) $$\frac{\alpha_{hnf}}{\alpha_{f}}P_{2}=\frac{P_{1}}{2}\frac{\cos(m)-\cosh(m)-\sin(m)\sinh(m)}{\cos(m)-\cosh(m)+\sin(m)\sinh(m)}.$$

**Figure 2:**
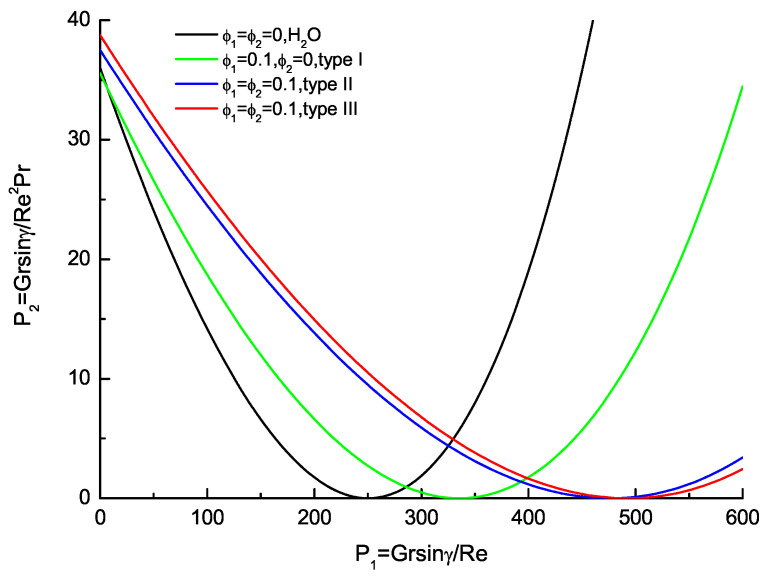
Flow regime map of the base fluid ($H_{2}O$), nanofluid (Cu-$H_{2}O$,type I) and hybrid nanofluid (Cu-$Al_{2}O_{3}$-$H_{2}O$,type II and III).

The flow regime map of the base fluid ($H_{2}O$), nanofluid (Cu-$H_{2}O$,type I) and hybrid nanofluid (Cu-$Al_{2}O_{3}$-$H_{2}O$,type II and III) with $\phi_{1},\phi_{2}$, the inflexion exists for each curve with corresponding to $P_{2}=0$ and $P_{1}=P_{1,\;c}$ is shown in Figure 2 and Table 5. About the base fluid, nanofluid and hybrid nanofluid, $P_{1,\;c}$ is the critical value for $P_{1}$ which value is 250.281948 (Lavine’s case [7]) for $\phi_{1}=\phi_{2}=0$. For $P_{1}\leq P_{1,\;c}$, the involved region is divided into two parts by a certain curve. The upper part is the regime that the flow reversal occurs to the top wall only, and the lower part is the regime in which no flow reversal can be measured. For $P_{1}\geq P_{1,\;c}$, this curve separates the regime for which flow reversal occurs to both walls of the regime of flow reversal of the bottom wall only. For a vertical channel ($P_{2}$ = 0), flow reversal must occur to both walls of the flow in symmetric about the channel centerline in this configuration. About the nanofluid, $P_{1,\;c}=288.675450$ for $\phi_{1}=0.05$ and $P_{1,\;c}=335.346949$ for $\phi_{1}=0.1$. Furthermoer, the hybrid nanofluid, $P_{1,\;c}=342.496247$ (type II) and $P_{1,\;c}=345.142372$ (type III) for $\phi_{1}=\phi_{2}=0.05$; $P_{1,\;c}=476.114704$ (type II) and $P_{1,\;c}=492.724576$ (type III) for $\phi_{1}=\phi_{2}=0.1$, respectively. For a horizonal channel ($P_{1}=0$), it is expected that the occurrence of flow reversal of upper wall is founded for $P_{2}>P_{2,\;c}$, where $P_{2,\;c}$ is the critical value of $P_{2}$ with its value. With $\phi_{1}=\phi_{2}=0$, $P_{2,\;c}=35.999978$ with the base fluid, $P_{2,\;c}=35.972464$ with the nanofluid and hybrid nanofluid. About nanofluid, $P_{2,\;c}=35.543514$ for $\phi_{1}=0.05$ and $P_{2,\;c}=35.562942$ for $\phi_{1}=0.1$. And hybrid nanofluid, $P_{2,\;c}=36.203632$ (type II) and $P_{2,\;c}=36.467267$ (type III) for $\phi_{1}=\phi_{2}=0.05$; $P_{2,\;c}=37.499401$ (type II) and $P_{2,\;c}=38.736813$ (type III) for $\phi_{1}=\phi_{2}=0.1$, respectively. With increasing from $\phi_{1},\phi_{2}$, the critical values $P_{1,\;c}$ enlarges simultaneously. Notice that $P_{1,\;c}$ enlarges on increasing $\phi_{1},\phi_{2}$, while $P_{2,\;c}$ also increases. Results show that the nanoparticle volume fractions $\phi_{1},\phi_{2}$ play a key role in delaying the occurrence of the flow reversal. The hybrid nanofluids hold more delayed range than conventional nanofluids, which is about 2.5 times that of nanofluids. The calculations of hybrid nanofluid about type II and type III agree very well; the relative error compared with hybrid nanofluid of type II is 0.7% for $\phi_{1}=\phi_{2}=0.05$ and 3% for $\phi_{1}=\phi_{2}=0.1$. So the hybrid nanofluid of type III that is proposed based on the linear assumptions is useful.

**Figure 3:**
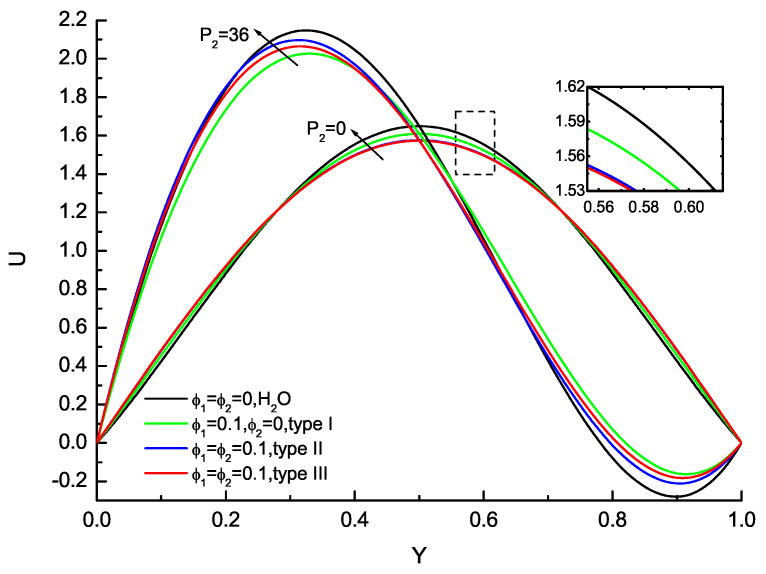
Variation with $P_{2}$ of the velocity profiles for the base fluid ($H_{2}O$), nanofluid (Cu-$H_{2}O$, type I) and hybrid nanofluid (Cu-$Al_{2}O_{3}$-$H_{2}O$, type II & III) at $P_{1}$ = 100.

**Figure 4:**
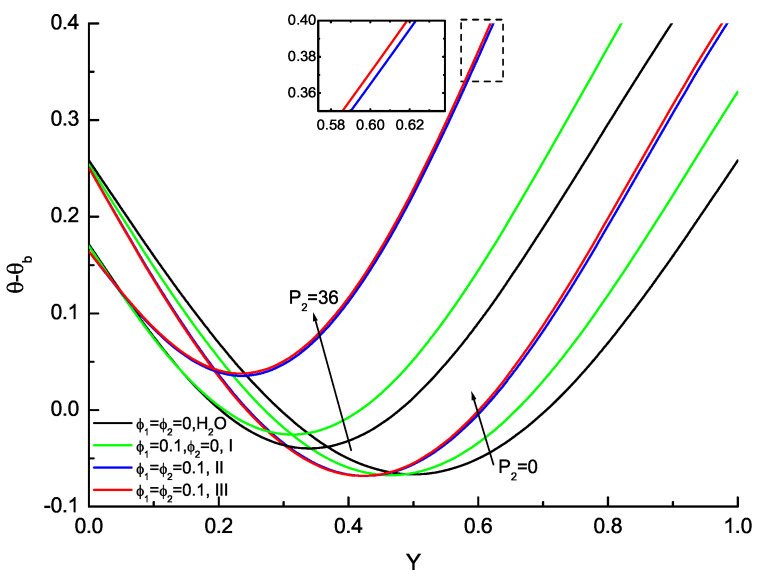
Variation with $P_{2}$ of the temperature profiles for the base fluid ($H_{2}O$), nanofluid (Cu-$H_{2}O$, type I) and hybrid nanofluid (Cu-$Al_{2}O_{3}$-$H_{2}O$, type II & III) at $P_{1}$ = 100.

Velocity and temperature profiles for the base fluid, nanofluid and hybrid nanofluid are presented in Figure 3 and Figure 4. The velocity and temperature profiles for the liquid at $P_{1}$=100 with $P_{2}=0$ and $P_{2}=36$ have been analyzed and temperature profiles will be illustrated as $\theta -\theta_{b}$ that eliminate the *x*-dependence. The velocity profiles are symmetric about the centerline and no flow reversals are found for all considered $\phi_{1},\phi_{2}$ for $P_{2}=0$ corresponding to the vertical channel configuration. The velocity near the walls increases as $\phi_{1},\phi_{2}$ increase, while the velocity in the vicinity of the centerline decreases from $\phi_{1},\phi_{2}$ increasing. The effects of $\phi_{1},\phi_{2}$ on the velocity distribution are evident; the increases in $\phi_{1},\phi_{2}$ delay the velocity reduction near the upper wall compared with the base fluid, nanofluid and hybrid nanofluid. Furthermore, the calculations of hybrid nanofluid about type II and type III agree very well. In the case of $P_{2}=36$, for any given values of $\phi_{1},\phi_{2}$, it is found that the velocity peak enlarges in magnitude and moves to the lower wall (Y=0) compared with $P_{2}=0$, while the velocity near the upper wall (Y=1) deceases with the flow reversal. The temperature profiles for the base fluid, nanofluid and hybrid nanofluid at $P_{1}=100$ are presented in Figure 4. The temperature near the upper wall increases accordingly as $P_{2}$ enlarges. At the same time, the valley of the temperature shifts towards the lower wall and its value increases consecutively with the increase in $P_{2}$. The temperature variation near the upper wall becomes larger and larger and the valley of the temperature moves to the upper wall gradually as $\phi_{1},\phi_{2}$ increase for $P_{2}$. The hybrid nanofluids about type II and type III agree very well, which are potential fluids that offer better thermophysical properties and heat transfer performance than convectional fluids and nanofluids with single nanoparticles.

**Figure 5:**
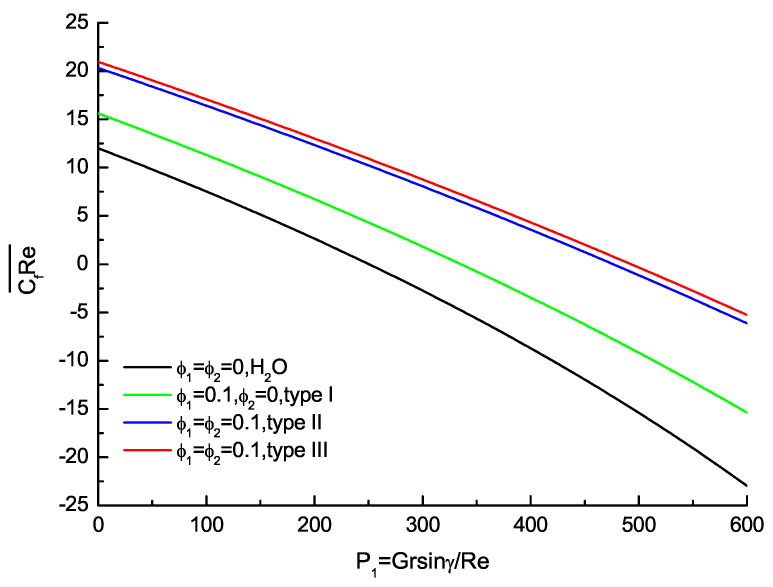
Variation of average $C_{f}Re$ with $P_{1}$ when Re=10,Pr=1 and Gr=3000.

**Figure 6:**
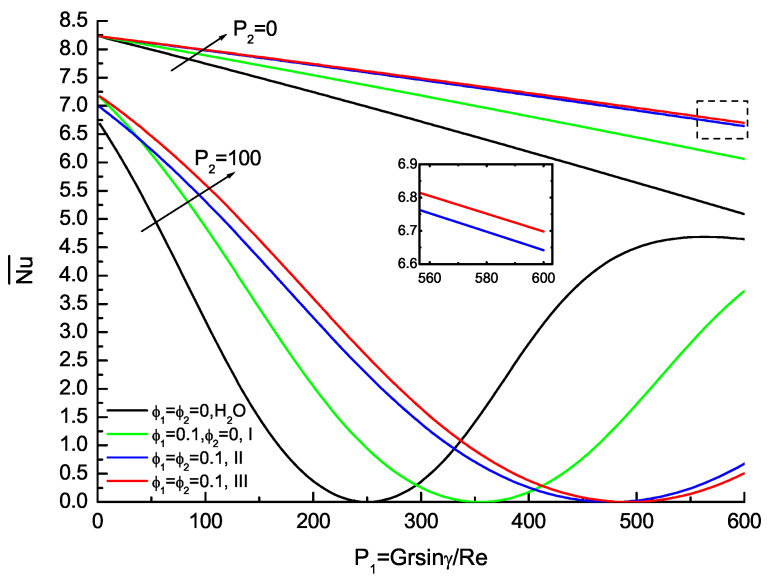
Variation of average Nu with $P_{1}$ for $P_{2}=0$ and $P_{2}=100$ when Re=10,Pr=1 and Gr=3000.

The average wall friction $\bar{C_{f}Re}$ and the average Nusselt number $\bar{Nu}$ are also physical quantities of practical interests, as shown as Figure 5 and Figure 6. Figure 5 shows the effects of $\phi_{1},\phi_{2}$ on the average wall friction with the base fluid, nanofluid and hybrid nanofluid. The average wall friction that is the independent of parameter $P_{2}$ decreases monotonously as $P_{1}$ increases. The hybrid nanofluids about type II and type III agree very well as $P_{1}$ increases, and the relative error compared with hybrid nanofluid of type II is 3% for $\phi_{1}=\phi_{2}=0.1$. Figure 6 shows the effects of $\phi_{1},\phi_{2}$ on the average Nusselt number with the base fluid, nanofluid and hybrid nanofluid. Contrary to the average friction, the average Nusselt number is dependent of $P_{2}$ and its value is equal to the average of top and bottom Nusselt number for $P_{1},P_{2}$ and $\phi_{1},\phi_{2}$ are prescribed. The average Nusselt number increases monotonously with the base fluid, nanofluid and hybrid nanofluid as $P_{1}$ increases for $P_{2}=0$. At the same time, the valley of the average Nusselt number shifts towards the lower wall and its value increases consecutively with the increase in $P_{2}$. The effects of $\phi_{1},\phi_{2}$ on the average Nusselt number distribution are evident with the base fluid, nanofluid and hybrid nanofluid. Furthermore, the calculations of hybrid nanofluid about type II and type III are in very good agreement. The hybrid nanofluids are potential fluids that offer better thermophysical properties and heat transfer performance than convectional fluids and nanofluids with single nanoparticles.

## 4. Conclusions

In this paper, fully developed mixed convection flow in the inclined channel filled by a hybrid nanofluid with a uniform wall heat flux boundary condition has been studied. The governing ordinary differential equations are made nondimensional and they are solved analytically. The explicitly analytical solutions of the velocity, temperature and pressure have been given. The effects of flow reversal, wall skin friction and Nusselt number of the hybrid nanofluid that depend on the nanoparticle volume fractions and pressure parameters are discussed and shown graphically (see Section 3). The hybrid nanofluids are potential fluids that offer better thermophysical properties and heat transfer performance than convectional fluids and nanofluids with single nanoparticles. The main key findings are listed below.

(1) The nanoparticle volume fractions play a key role in delaying the occurrence of the flow reversal. The hybrid nanofluids hold a more delayed range than conventional nanofluids, which is about 2.5 times that of nanofluids.
(2) Two hybrid models (type II and type III) are compared with different nanoparticle volume fractions $\phi_{1},\phi_{2}$ and pressure parameters $P_{1},P_{2}$. It is observed that they are in very good agreement, with the relative error less than 3%. The hybrid model of type III is useful and simplified in that it omits the nonlinear terms due to the interaction of different nanoparticle volumetric fractions.
(3) The effects of nanoparticle volume fractions on the velocity and temperature distributions are evident, the increases of $\phi_{1},\phi_{2}$ delay the velocity reduction and enlarge the temperature variation near the upper wall (Y=1) compared with the base fluid, nanofluid and hybrid nanofluid.
(4) The average wall friction ($\bar{C_{f}Re}$) that is independent of $P_{2}$ decreases monotonously as $P_{1}$ increases. The average Nusselt number ($\bar{Nu}$) that is dependent of $P_{2}$ increases monotonously as $P_{1}$ increases for the vertical channel ($P_{2}=0$). The valley of the average Nusselt number shifts towards the lower wall and its value increases consecutively with the increase of $P_{2}$.

## Figures and Tables

**Table 1:** Thermophysical properties of nanoparticles and water [33].

Physical Properties	Cu	${Al}_{2}O_{3}$	$H_{2}O$
ρ (kg/$m^{3}$)	8933	3970	997.1
$C_{p}$ (J/kg K)	385	765	4179
*k* (W/mK)	400	40	0.613
$\alpha \times 10^{7}$ ($m^{2}$/s)	11,163.1	131.7	1.47
$\beta \times 10^{-5}$ (1/K)	1.67	0.85	21

**Table 2:** Thermophysical properties of nanofluid (type I) [34].

Properties	Nanofluid
Density	$\rho_{nf}=\left(1-\phi_{1}\right)\rho_{f}+\phi_{1}{\left(\rho \beta \right)}_{n1}$
Thermal expansion coefficient	${\left(\rho \beta \right)}_{nf}=\left(1-\phi_{1}\right){\left(\rho \beta \right)}_{f}+\phi_{1}{\left(\rho \beta \right)}_{n1}$
Heat capacity	${\left(\rho C_{p}\right)}_{nf}=\left(1-\phi_{1}\right){\left(\rho C_{p}\right)}_{f}+\phi_{1}{\left(\rho C_{p}\right)}_{n1}$
Viscosity	$\mu_{nf}=\frac{\mu_{f}}{{\left(1-\phi_{1}\right)}^{2.5}}$
Thermal diffusivity	$\alpha_{nf}=\frac{k_{nf}}{{\left(\rho C_{p}\right)}_{nf}}$
Thermal conductivity	$\frac{k_{nf}}{k_{f}}=\frac{k_{n1}+2k_{f}-2\phi_{1}\left(k_{f}-k_{n1}\right)}{k_{n1}+2k_{f}+\phi_{1}\left(k_{f}-k_{n1}\right)}$
Electrical conductivity	$\frac{\sigma_{nf}}{\sigma_{f}}=1+\frac{3\left(\frac{\sigma_{n1}}{\sigma_{f}}-1\right)\phi_{1}}{2+\frac{\sigma_{n1}}{\sigma_{f}}-\left(\frac{\sigma_{n1}}{\sigma_{f}}-1\right)\phi_{1}}$

**Table 3:** Thermophysical properties of hybrid nanofluid (type II) [35].

Properties	Nanofluid
Density	$\rho_{hnf}=\left(1-\phi_{2}\right)\left[\left(1-\phi_{1}\right)\rho_{f}+\phi_{1}\rho_{n1}\right]+\phi_{2}\rho_{n2}$
Thermal expansion coefficient	${\left(\rho \beta \right)}_{hnf}=\left(1-\phi_{2}\right)\left[\left(1-\phi_{1}\right){\left(\rho \beta \right)}_{f}+\phi_{1}{\left(\rho \beta \right)}_{n1}\right]+\phi_{2}{\left(\rho \beta \right)}_{n2}$
Heat capacity	${\left(\rho C_{p}\right)}_{hnf}=\left(1-\phi_{2}\right)\left[\left(1-\phi_{1}\right){\left(\rho C_{p}\right)}_{f}+\phi_{1}{\left(\rho C_{p}\right)}_{n1}\right]+\phi_{2}{\left(\rho C_{p}\right)}_{n2}$
Viscosity	$\mu_{hnf}=\frac{\mu_{f}}{{\left(1-\phi_{1}\right)}^{2.5}{\left(1-\phi_{2}\right)}^{2.5}}$
Thermal diffusivity	$\alpha_{hnf}=\frac{k_{hnf}}{{\left(\rho C_{p}\right)}_{hnf}}$
Thermal conductivity	$\frac{k_{hnf}}{k_{nf}}=\frac{k_{n2}+2k_{nf}-2\phi_{2}\left(k_{nf}-k_{n2}\right)}{k_{n2}+2k_{nf}+\phi_{2}\left(k_{nf}-k_{n2}\right)}$, where $\frac{k_{nf}}{k_{f}}=\frac{k_{n1}+2k_{f}-2\phi_{1}\left(k_{f}-k_{n1}\right)}{k_{n1}+2k_{f}+\phi_{1}\left(k_{f}-k_{n1}\right)}$
Electrical conductivity	$\frac{\sigma_{hnf}}{\sigma_{nf}}=\frac{\sigma_{n2}+2\sigma_{nf}-2\phi_{2}\left(\sigma_{nf}-\sigma_{n2}\right)}{\sigma_{n2}+2\sigma_{nf}+\phi_{2}\left(\sigma_{nf}-\sigma_{n2}\right)}$, where $\frac{\sigma_{nf}}{\sigma_{f}}=\frac{\sigma_{n1}+2\sigma_{f}-2\phi_{1}\left(\sigma_{f}-\sigma_{n1}\right)}{\sigma_{n1}+2\sigma_{f}+\phi_{1}\left(\sigma_{f}-\sigma_{n1}\right)}$

**Table 4:** Thermophysical properties of hybrid nanofluid (type III) [36,37].

Properties	Nanofluid
Density	$\rho_{hnf}=\left(1-\phi_{1}-\phi_{2}\right)\rho_{f}+\phi_{1}\rho_{n1}+\phi_{2}\rho_{n2}$
Thermal expansion coefficient	${\left(\rho \beta \right)}_{hnf}=\left(1-\phi_{1}-\phi_{2}\right){\left(\rho \beta \right)}_{f}+\phi_{1}{\left(\rho \beta \right)}_{n1}+\phi_{2}{\left(\rho \beta \right)}_{n2}$
Heat capacity	${\left(\rho C_{p}\right)}_{hnf}=\left(1-\phi_{1}-\phi_{2}\right){\left(\rho C_{p}\right)}_{f}+\phi_{1}{\left(\rho C_{p}\right)}_{n1}+\phi_{2}{\left(\rho C_{p}\right)}_{n2}$
Viscosity	$\mu_{hnf}=\frac{\mu_{f}}{{\left(1-\phi_{1}-\phi_{2}\right)}^{2.5}}$
Thermal diffusivity	$\alpha_{hnf}=\frac{k_{hnf}}{{\left(\rho C_{p}\right)}_{hnf}}$
Thermal conductivity	$\frac{k_{hnf}}{k_{nf}}=\frac{k_{n2}+2k_{nf}-2\phi_{2}\left(k_{nf}-k_{n2}\right)}{k_{n2}+2k_{nf}+\phi_{2}\left(k_{nf}-k_{n2}\right)}$, where $\frac{k_{nf}}{k_{f}}=\frac{k_{n1}+2k_{f}-2\phi_{1}\left(k_{f}-k_{n1}\right)}{k_{n1}+2k_{f}+\phi_{1}\left(k_{f}-k_{n1}\right)}$
Electrical conductivity	$\frac{\sigma_{hnf}}{\sigma_{nf}}=\frac{\sigma_{n2}+2\sigma_{nf}-2\phi_{2}\left(\sigma_{nf}-\sigma_{n2}\right)}{\sigma_{n2}+2\sigma_{nf}+\phi_{2}\left(\sigma_{nf}-\sigma_{n2}\right)}$, where $\frac{\sigma_{nf}}{\sigma_{f}}=\frac{\sigma_{n1}+2\sigma_{f}-2\phi_{1}\left(\sigma_{f}-\sigma_{n1}\right)}{\sigma_{n1}+2\sigma_{f}+\phi_{1}\left(\sigma_{f}-\sigma_{n1}\right)}$

**Table 5:** The critical values of $P_{1}$ and $P_{2}$.

Critical Values	Types	$\phi_{1}=\phi_{2}=0$	$\phi_{1}=\phi_{2}=0.05$	$\phi_{1}=\phi_{2}=0.1$
$P_{1,\;c}$	$H_{2}O$ (Lavine’s case [7])	250.281948	-	-
	Cu-$H_{2}O$ (type I)	250.281948	288.675450	335.346949
	$Al_{2}O_{3}$-$H_{2}O$ [12]	250.281948	296.981071	355.527252
	Cu-$Al_{2}O_{3}$-$H_{2}O$ (type II)	250.281948	342.496247	476.114704
	Cu-$Al_{2}O_{3}$-$H_{2}O$ (type III)	250.281948	345.142372	492.724576
$P_{2,\;c}$	$H_{2}O$ (Lavine’s case [7])	35.999978	-	-
	Cu-$H_{2}O$ (type I)	35.972464	35.543514	35.562942
	$Al_{2}O_{3}$-$H_{2}O$ [12]	35.972464	36.597696	37.750800
	Cu-$Al_{2}O_{3}$-$H_{2}O$ (type II)	35.972463	36.203632	37.499401
	Cu-$Al_{2}O_{3}$-$H_{2}O$ (type III)	35.972463	36.467267	38.736813

## Data Availability

All drawings were made by myself.

## Nomenclature

The following nomenclature is used in this manuscript:

A,B	Constants
$C_{f}$	Wall skin friction coefficient
${\bar{C}}_{f}$	Average wall skin friction
$C_{p}$	Specific heat at constant pressure
*g*	Acceleration due to gravity
Gr	Grashof number
$k_{hnf}$	Thermal conductivity of the hybrid nanofluid
$k_{f},k_{n}$	Thermal conductivities of the base fluid and nanofluid, respectively
*L*	Distance between the walls
Nu	Nusselt number
*p*	Dimensional thermodynamic pressure
*P*	Dimensionless thermodynamic pressure
$P_{1},P_{2}$	Dimensionless pressure parameters
$P_{1,c},P_{2,c}$	Critical values for $P_{1}$ and $P_{2}$, respectively
Pr	Prandtl number
*q*	Constant wall heat flux
Re	Reynolds number
*T*	Nanofluid temperature
$T_{b},T_{w}$	Bulk and wall temperature, respectively
$T_{0}$	Constant reference temperature
u(y)	Dimensional velocity component along the *x* axis
$\bar{u}$	Dimensional average velocity
U(Y)	Dimensionless velocity component along the *X* axis
*x*	Dimensional axis measured along lower plane of channel oriented in downward direction
*y*	Dimensional axis measured in the normal direction to the lower plane
X,Y	Dimensionless coordinates
$\alpha_{hnf}$	Thermal diffusivity of the hybrid nanofluid
$\alpha_{f}$	Thermal diffusivity of the base fluid
$\beta_{f},\beta_{n}$	Coefficients of thermal expansion of the fluid and nanofluid, respectively
$\phi_{1},\phi_{2}$	Nanoparticle volume fractions
γ	Inclination of the channel
$\mu_{hnf}$	Viscosity of the hybrid nanofluid
$\mu_{f}$	Dynamic viscosity of the base fluid
$\nu_{f}$	Kinematic viscosity of the base fluid
θ	Dimensionless nanofluid temperature
$\theta_{b},\theta_{w}$	Dimensionless bulk and wall temperature, respectively
${\left(\rho C_{p}\right)}_{hnf}$	Heat capacitance of the hybrid nanofluid
$\rho_{f},\rho_{n}$	Densities of the fluid and nanofluid fractions, respectively
$\tau_{w}$	Skin friction or wall shear stress
